# Patterns of Cannabis Use Among US Middle-Aged and Older Adult Cannabis Users

**DOI:** 10.1192/j.eurpsy.2023.84

**Published:** 2023-07-19

**Authors:** O. Livne

**Affiliations:** ^1^ New York State Psychiatric Institute; ^2^Department of Epidemiology, Mailman School of Public Health, Columbia University, New York, United States

## Abstract

**Abstract:**

Cannabis use is sharply increasing among middle-aged and older US adults, two populations that are particularly vulnerable to the detrimental effects of cannabis use. In recent decades patterns of cannabis use (e.g., method of consumption, product type, and potency) have become increasingly heterogeneous. However, little is known about the differences in such patterns between younger adult, middle-aged, and older adult users.

In this presentation, we will provide clinicians and researchers with important information on a wide array of patterns of cannabis use among adults ages ≥50 years, and highlight potential risks and harm reduction strategies. Findings from a recent study will be presented. Respondents were 4,151 US adult past 7-day cannabis users who participated in an online survey administered via social media platforms. Using logistic and linear regression models, we examined whether middle-aged (50-64 years; n=1,080), and older adult (≥65 years; n=295) respondents differed from younger (18-49 years; n=2,776) respondents, and from each other across several patterns of cannabis use. Results show that in comparison with younger adults, middle-aged and older adults were more likely to consume cannabis products earlier during the day, by fewer methods of consumption, exclusively by smoking, and in smaller amounts, but were less likely to consume cannabis products that are highly potent, and by methods of consumption other than smoking. Significant differences were also observed in several patterns of cannabis use between older and middle-aged adults, including time of day of use, methods of consumption, potency and amounts of use.

In a changing cannabis use landscape, our findings indicate that middle-aged and older adults may be less affected by the recently increasing heterogeneity in patterns of cannabis use, but also inform on the need for targeted harm reduction approaches. Findings also highlight existing gaps in the literature and future research directions.

**Disclosure of Interest:**

None Declared

